# Supplementary material for: Hierarchical semantic composition of biosimulation models using bond graphs
Source: PLoS Comput Biol. 2021 May 13;17(5):e1008859. doi: 10.1371/journal.pcbi.1008859 (PMC8148364; doi:10.1371/journal.pcbi.1008859)
Supplement: S1 Table — The fitting options which are not mentioned in the table are remained untouched as their default values in the MATLAB fitting tool. (PDF) [file pcbi.1008859.s001.pdf]

	Parameter	Value/Option	Description
Function coefficients	a_1	0.0005422	
	b_1	0.1317	
	c_1	0.03315	
	a_2	0.0003591	
	b_2	0.181	
	c_2	0.06554	
Fit options	$DiffMinChange$	$1.0e^{-3}$	
	$DiffMaxChange$	0.1	
	$Robust$	LAR	Minimizing the Least Absolute Residuals.
	$Algorithm$	$Levenberg - Marquardt$	If the fit generated by the trust-region algorithm is not acceptable, try Levenberg-Marquardt.
	$MaxFunEvals$	60,000	Maximum number of allowed evaluations for the selected function.
	$TolFun$	$1.0e^{-9}$	Termination tolerance in stopping conditions for the function.
	$TolX$	$1.0e^{-10}$	Termination tolerance in stopping conditions for the coefficients.
