# Supplementary figures and images for: Hierarchical semantic composition of biosimulation models using bond graphs
Source: PLoS Comput Biol. 2021 May 13;17(5):e1008859. doi: 10.1371/journal.pcbi.1008859 (PMC8148364; doi:10.1371/journal.pcbi.1008859)

Blood flow (cm<sup>3</sup>/s)

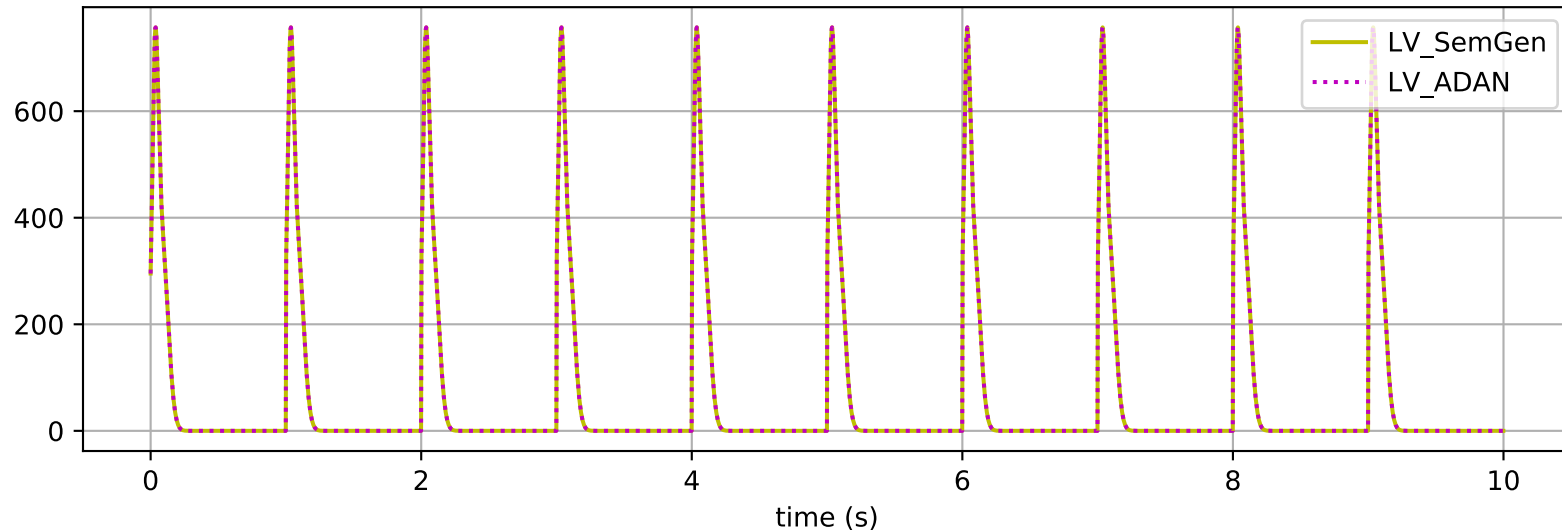

Supplement: S1 Fig — The yellow line and dashed red line show the same imposed flow in the SemGen merged model and the ADAN open-loop model. (PDF) [file pcbi.1008859.s002.pdf]
