# Supplementary material for: Hierarchical semantic composition of biosimulation models using bond graphs
Source: PLoS Comput Biol. 2021 May 13;17(5):e1008859. doi: 10.1371/journal.pcbi.1008859 (PMC8148364; doi:10.1371/journal.pcbi.1008859)
Supplement: Attachment — Submitted filename: reviewers response letter.pdf [file pcbi.1008859.s003.pdf]

Dr. Daniel A Beard

Deputy Editor

PLOS Computational Biology

April 24, 2021

Subject: Submission of revised paper PCOMPBIOL-D-21-00472

Dear Dr. Beard,

Thank you for your email dated 15 April 2021 enclosing the reviewers' comments, and for giving us the opportunity to submit a revised draft of our manuscript titled "Hierarchical semantic composition of biosimulation models using bond graphs" for publication in *PLOS Computational Biology*. We have incorporated changes to reflect the suggestions and comments provided by the reviewers. We have highlighted the changes within the manuscript in a point-by-point manner below.

Comment to the editor by one of the reviewers:

Briefly review other software modeling tools that adopt the bond graph formalism.

> **Response:** Thank you for pointing this out and we have, accordingly, added a paragraph to the Introduction section to cover this point. The relevant paragraph is located on page 4 of the manuscript and is highlighted in blue.

Reviewer 2:

Comment #1: *It would be interesting for the readers to discuss some aspects regarding the use of entire arterial network (i.e., including the cerebral system). Which are the difficulties and problems that could arise when we deal with the cerebral system?*

> **Response:** We agree with this and have incorporated your suggestion throughout the manuscript. The expansion of our approach to include the cerebral system along with the required modifications have been discussed in the Discussion section on pages 14-15 and is highlighted in green.

Reviewer 2:

Comment #2: *The overall model is based on lumped-parameter models. There are some losses when we use this kind of model instead of PDEs description? Is there a workable solution with bond graphs for biological systems modelled via PDEs?*

> **Response:** Thank you for raising this point here. Bond graphs are primarily an approach to lumped parameter modelling, as noted by the Reviewer. Thus a discussion of the relative merits of lumped parameter ODE models vs spatially extended PDE models is indeed germane to the benefits and limitations of the bond graph approach. Bond graphs can not capture PDE type models. There is, however, a natural extension of the port-based bond graph approach to modelling to incorporate spatially extended systems. This approach, called port-Hamiltonian theory, provides a generalized formulation for network modeling of multi-physics systems. A brief discussion has been added on page 15 and is highlighted in yellow.

We hope the revised version is now suitable for publication and look forward to hearing from you in due course.

Sincerely,

Niloofar Shahidi (on behalf of the authors)

Auckland Bioengineering Institute

University of Auckland

E-mail: nsha457@aucklanduni.ac.nz
